# Enhanced Keratinocyte Proliferation and Migration in Co-culture with Fibroblasts

**DOI:** 10.1371/journal.pone.0040951

**Published:** 2012-07-20

**Authors:** Zhenxiang Wang, Ying Wang, Farhang Farhangfar, Monica Zimmer, Yongxin Zhang

**Affiliations:** 1 Department of Plastic and Aesthetic Surgery, Southwest Hospital, Third Military Medical University, Chongqing, People’s Republic of China; 2 Department of R&D, ZYX Biotech Company, Carrollton, Texas, United States of America; 3 Regenetech Inc., Houston, Texas, United States of America; Catholic University Medical School, Italy

## Abstract

Wound healing is primarily controlled by the proliferation and migration of keratinocytes and fibroblasts as well as the complex interactions between these two cell types. To investigate the interactions between keratinocytes and fibroblasts and the effects of direct cell-to-cell contact on the proliferation and migration of keratinocytes, keratinocytes and fibroblasts were stained with different fluorescence dyes and co-cultured with or without transwells. During the early stage (first 5 days) of the culture, the keratinocytes in contact with fibroblasts proliferated significantly faster than those not in contact with fibroblasts, but in the late stage (11^th^ to 15^th^ day), keratinocyte growth slowed down in all cultures unless EGF was added. In addition, keratinocyte migration was enhanced in co-cultures with fibroblasts in direct contact, but not in the transwells. Furthermore, the effects of the fibroblasts on keratinocyte migration and growth at early culture stage correlated with heparin-binding EGF-like growth factor (HB-EGF), IL-1α and TGF-β1 levels in the cultures where the cells were grown in direct contact. These effects were inhibited by anti-HB-EGF, anti-IL-1α and anti-TGF-β1 antibodies and anti-HB-EGF showed the greatest inhibition. Co-culture of keratinocytes and IL-1α and TGF-β1 siRNA-transfected fibroblasts exhibited a significant reduction in HB-EGF production and keratinocyte proliferation. These results suggest that contact with fibroblasts stimulates the migration and proliferation of keratinocytes during wound healing, and that HB-EGF plays a central role in this process and can be up-regulated by IL-1α and TGF-β1, which also regulate keratinocyte proliferation differently during the early and late stage.

## Introduction

Wound repair and scar formation are complex and important processes in clinical care. Scar formation characterized by hypertrophy, contracture, and scar instability significantly impairs late functional and aesthetic outcomes [1]. Conventional split-thickness skin grafts, which are often widely meshed and expanded, are utilized to close large wound deficits. The consequences of scarring and contracture often require lengthy courses of revisional surgery [1]. Tissue repair is divided into an inflammatory phase, a granulation phase with synthesis of new connective tissue and epithelial wound closure, and finally a scar remodeling phase once the epidermal barrier has been re-established. In the mid- and late phases of wound healing, cellular interactions become dominated by the interplay of keratinocytes with fibroblasts, which gradually shift the microenvironment away from an inflammatory to a synthesis-driven granulation phase [2].

Untreated full-thickness wounds often heal with marked contracture and a deforming scar. It is well accepted that a wound that takes longer than 2–3 weeks to heal is at an increased risk of hypertrophic scar formation and contracture [3]. This risk can be reduced by the timely application of a split-thickness skin graft [4], and the application of a split-thickness skin graft or a dermal substitute to a full-thickness wound also inhibits the degree of contraction. This finding has been correlated with a reduction in inflammatory infiltrate [3]. The grafting of granulating wounds results in a rapid decrease in the number of myofibroblasts mediated by an apoptosis mechanism. However, graft contraction and hypertrophic scar formation remains a considerable problem even when skin grafts are applied in a timely manner. It has been estimated that more than 20% of patients with burns will develop significant hypertrophic scarring or graft contracture [3]. Therefore, we hypothesized that other factors, such as the interaction between keratinocytes and fibroblasts, may also significantly affect the wound healing process.

Wound healing is dependent on the recruitment of several cell types that appear in the wound area in a temporally- and spatially-defined manner [5]. Reepithelialization largely coincides with the recruitment of dermal fibroblasts, and it is likely that crosstalk between epidermal keratinocytes and fibroblasts is important during the rebuilding of tissue integrity [6]. The healing of extensive wounds often results in excessive scarring, disgorging, and functional impairment of the affected area [7]. This is a particular problem in the healing of large burn wounds, and it appears that early reepithelialization or coverage of the wounded area with autologous skin grafts limits excessive deposition of connective tissue. Experiments where keratinocytes were co-cultured with fibroblasts have demonstrated the establishment of paracrine loops of cytokines between the two cell types [8], [9], which is a phenomenon that may also occur *in vivo* to regulate cellular function [10]. It has been suggested that epidermal keratinocytes can down-regulate the production of the major matrix component of the dermis, collagen I, by fibroblasts [11], [12]. However, other studies have demonstrated that keratinocytes can be expanded by co-culturing them with human dermal fibroblasts (fibroblasts) without fetal calf serum [13]. It has also been shown that human fibroblasts can support the expansion of keratinocytes, and keratinocytes that were expanded on fibroblasts in the absence of serum tended to exhibit less differentiation than those expanded with serum [13]. To further understand these conflicting research results, the interaction of keratinocytes and fibroblasts in different culture stages and the molecular mechanisms involved were explored in this study.

## Materials and Methods

### Culture Media

Several different combinations of hormones and growth factors were used in our studies. For the cultivation of fibroblasts, we used RPMI1640 medium containing 10% (v/v) fetal bovine serum (FBS, Gibco-BRL, Germany), 100 U/mL penicillin, and 100 µg/mL streptomycin (pH 7.2) (Gibco-BRL, Germany). For the cultivation of keratinocytes, keratinocyte growth medium (KGM, serum-free medium, Provitro, Germany) was used with its supplements but containing no EGF except where otherwise indicated. For the stimulation mimic tests, EGF (R&D Systems, US), Recombinant human heparin-binding EGF-like growth factor (HB-EGF) (R&D Systems), IL-1α (R&D Systems), or TGF-β1 (R&D Systems) were added to KGM at a concentration of 0, 10, 20 or 40 ng/ml, respectively. Goat anti-human HB-EGF polyclonal antibody, mouse anti-IL1α monoclonal antibody, and mouse anti-TGF-β1 monoclonal antibody and their isotype controls were all purchased from Biocompare, South San Francisco, CA, USA and used at a concentration of 0.5 µl/ml for the corresponding cytokine neutralization tests. For the validation of the tests, the test values from all 4 isotype controls must be in the range of 1× standard deviation (±SD) of the means from 4 blank controls. Collagenase type II, Trypsin/EDTA, and phosphate-buffered saline (PBS) (0.15 M) were obtained from Gibco-BRL, Germany. Collagen type I from bovine skin was purchased from Sigma, USA.

### Isolation and Culture of Human Dermal Fibroblasts

Human fibroblasts and keratinocytes were obtained from the dermis of juvenile human foreskin at the time of circumcision, with consent from the parents, as previously described [14], [15]. Samples were transferred to RPMI medium containing 10% (v/v) FBS, 100 U/mL penicillin, and 100 µg/mL streptomycin and stored for up to 1 week at 4°C. Samples were washed three times in RPMI containing penicillin-streptomycin and Nystatin to remove possible pathogens and then rinsed several times with PBS to remove any blood and serum. Most of the subcutaneous fat and membranous material was removed with either a scalpel blade or a sharp pair of curved scissors. The skin was cut into strips 1 cm wide using a scalpel and the epidermis was separated from the dermis using two pairs of forceps.

For the separation of keratinocytes, the strips were placed, epidermis side up, in 0.25% trypsin and then incubated at 4°C overnight or at 37°C for 1 h. The dermis was cut into very small pieces (5 mm^2^) using curved scissors and the pieces were transferred into a collagenase Type II (200 IU/ml) solution in an incubator at 37°C for 2 h. The suspension was then centrifuged at 500×g for 5 min. The pellet was resuspended in culture medium. The isolated cells were counted using a hemocytometer and then seeded into culture dishes at a cell density of 2×10^5^ cells/cm^2^. The cells were cultured in a CO_2_ incubator at 37°C and the culture medium was changed twice a week. The cells were passaged before reaching confluence by treating them with Trypsin-EDTA for 5 min, and then split into new dishes at a 1∶3 dilution [9], [14].

### Isolation and Culture of Human Epidermal Keratinocytes

The epidermis obtained from the previous section was incubated in 0.25% trypsin solution at 4°C overnight or at 37°C for 1 h. The trypsinized tissue was transferred into a 15 ml centrifuge tube and vortexed gently for 2 min followed by the addition of an equal volume of FBS to inhibit the action of the trypsin. The suspension was centrifuged at 400×g for 5 min, the supernatant was discarded, and the cell pellet was resuspended in KGM culture medium. After counting the cells and determining the viability using the trypan blue exclusion method, the cells were seeded in tissue culture dishes coated with Collagen I/fibronectin/bovine serum albumin (BSA). Approximately 40% of the epidermal cells attached and began to spread out on the dish within 24–48 h. The culture medium was changed daily and the dishes were confluent by 7–10 d post-seeding [5], [8], [12].

### Staining with PKH2 and PKH26

Fibroblasts and keratinocytes were stained with PKH2 and PKH26, respectively (Sigma ImmunoChemicals, US), before being used in the cultures according to the manufacturer’s instructions. Briefly, cells were suspended in 1 mL of diluent and immediately transferred into polypropylene tubes containing 1 mL of 4 µM PKH2 or PKH26 in diluent at room temperature. After 5 min incubation with frequent agitation, 2 mL of FBS was added to the suspension followed by an additional incubation for 1 min. The total volume was brought up to 8 mL with complete medium (Iscove’s modified Dulbecco’s medium [IMDM] supplemented with 10% FBS, 1× L-glutamine, and antibiotics [100 U/mL penicillin and 100 µg/mL streptomycin]), and the cells were washed three times in complete medium. After centrifugation at 400×g for 5 min, the cell pellet was resuspended in PBS and transferred to a new tube. The cells were washed two more times in PBS in the same tube, and then resuspended in complete medium and seeded for short-term culture as described below. Keratinocytes stained with PKH2 at appropriate concentrations for the experiments were seeded in 6-well plates, transwell plates, or 100×20 cell culture dishes pre-coated with collagen I/fibronectin/BSA based on different culture purposes as described above. The next day, the cells were washed with PBS and conditioned for 3 h with 10 ml DMEM/Ham’s F12 (4∶1) supplemented with 0.5% FBS. The cell density was approximately 50% at this point.

### Co-culture Methods

#### Basic culture conditions

For basic culture conditions, 0.15 or 0.3×10^6^ fibroblasts were cultured per well of 6-well Falcon multi-well plates (pre-coated with Collagen I/fibronectin/BSA) with a surface area of 9.62 cm^2^ (BD Labware, Franklin Lakes, NJ) or in Falcon polyurethane cell culture inserts (upper chamber, 0.4 mm pore diameter) based on the requirements of the different experiments. After 48 h, 1.0 or 1.5×10^6^ keratinocytes were seeded on top of the fibroblasts or in the upper chamber of the cell culture insert. The inserts were pre-coated with a mixture of 10 mg/ml bovine plasma fibronectin (Gibco BRL/Life Technology, Paisley, U.K.), 30 mg/ml bovine collagen I (Vitrogen, Cohesion, Palo Alto, CA), and 10 mg/ml BSA (Sigma) for 2 h at 37°C. As controls, fibroblasts or keratinocytes were cultured individually at the same time at the corresponding concentrations. The culture medium used before the establishment of the co-culture was the standard growth medium for the two cell types as described above. Upon initiation of the co-cultures, the medium was changed to KGM with supplements but without EGF, except where otherwise indicated, depending on the different experiment designs. The total volume was 4 ml (2+2 ml).

#### Migration assay

All 6-well plates and coverslips were pre-coated with collagen I/fibronectin/BSA as described above. PKH2-stained fibroblasts (0.3×10^6^ cells) were seeded on coverslips at the center of the bottom compartment of 6-well plates and cultured overnight at 37°C and 5% CO_2_. The next day, the medium was removed and 5 ml of PKH26-stained keratinocytes were seeded into each well at a density of 1×10^6^ cells/well. In the K/Fp group, before keratinocytes were added, fibroblasts were treated with 1% paraformaldehyde for 5 minutes and washed 6 times with medium. After 48 hours, all cells were treated with Mytomycin C (10 µg/ml) for 3 hours, and washed 3 times to inhibit cell proliferation. Then, the coverslips were transferred into new wells, and 5 ml of new medium was added. At this time, some cultures received media containing anti-TGFβ1, anti-IL1α or anti HB-EGF. The cells were cultured for an additional 5 days, and then the coverslips were removed from the wells. The wells were washed with PBS and the cells were removed using a standard trypsin treatment procedure. The cells were resuspended in 0.5 ml PBS, counted, and then compared to cells from the wells where either fibroblasts or keratinocytes were not added or were added in the upper chambers of the Transwells. Each condition was tested in 4–6 replicate wells. The cell type and number were determined by Trypan Blue exclusion and Flow Cytometry. To validate our cover slip method, scratch assay, a classic method [16]–[20] for evaluating cell migration, was used for the correlation analysis in different concentrations of cells and HB EGF (0, 5, 10, 20 and 40/ng/ml) which resulted in different cell migration distances. Cells in scratch assay were also treated with Mitomycin C (10 ug/ml, 3 hours) and the migration distance was measured at 48 hours following culture. When 0.1, 0.3, 0.6 and 0.9×10^6^ fibroblasts and 0.5, 1.0, 1.5 and 2.0×10^6^ keratinocytes were respectively combined for the tests, the cell counts in cover slip assay and the cell moving distance in scratch assay at different time points correlated very well (R^2^>0.90, P<0.01). It can be concluded that the results of cover slip assay objectively reflect cell migration.

#### Proliferation test

This assay was similar to the migration test, but no cover slip, Mitomycin C and Paraformaldehyde were used. The cells in four wells of 6-well plates for each condition were harvested as described above after 5, 10 and 15 days of culture, respectively.

### Microscopy

The keratinocytes (stained with PKH26) and fibroblasts (stained with PKH2) were grown in 6-well plates and then processed as described above. Images were taken with an inverted microscope (ECLIPSE TE2000-U; Nikon) using a red and green Fluor 20x/0.45 objective (Nikon), regular light or natural light and analyzed with Image-Pro Plus software (Media Cybernetics).

### Flow Cytometry Analysis

Flow cytometry was performed on trypsinized keratinocytes as described by van Erp et al. (1988), and 1% BSA was added to the wash buffer. Briefly, the keratinocytes (stained with PKH26) and fibroblasts (stained with PKH2) grown in 6-well plates were trypsinized, washed with wash buffer (PBS containing 0.5% BSA), and then fixed with 4% paraformaldehyde in PBS. The cell population was gated to exclude cell debris and analyzed with FACS CellQuest software (BD, US). Fifteen thousand events were acquired in list mode according to the following four parameters: forward scatter, side scatter, green fluorescence, and red fluorescence. PKH2 or PKH26 positive cells were calculated with the formula: total cell number (from Trypan Blue exclusion cell counting) × PKH2 or PKH26 positive percentages (from flow cytometry).

Annexin V binding assay was performed by staining cells with Annexin V-PE contained in an apoptosis detection kit, according to the manufacturer’s instructions (BD Pharmingen, San Diego, CA, USA). Binding was assessed on day 5, day 10 and day 15 as in our previous studies [21].

### ELISA for EGF, HB-EGF, IL-1α and TGF-β1

Cell culture supernatants were harvested and EGF (R&D Systems), HB-EGF (Biocompare, US), IL-1α (Biocompare), and TGF-β1 (Biocompare) ELISA kits were used to detect the respective cytokines following the manufacturers’ instructions, which were similar to the procedures in our previous reports [22]–[25]. Briefly, 100 µl of the sample was added into each well of a pre-coated ELISA plate in triplicate. After the incubation and washes, the detection antibodies were added. Following additional incubation and wash steps, the substrates were added and color was developed. The absorbance in each well was read at a wavelength of 415 nm using an automatic microplate reader (Molecular Devices, Sunnyvale, CA). The data were collected using SOFTmax data reduction software (Molecular Devices). All assays were conducted with standards, and the concentrations of the above cytokines in the test samples were extrapolated from the standard curves and expressed as pg/ml.

### IL-1α and TGF-β1 siRNA Transfection

Fibroblasts were transfected with small interfering RNA (siRNA) using an Amaxa Nucleofector (Gaithersburg, MD) and Basic Nucleofector Kit for Mammalian Fibroblasts (Amaxa) according to the manufacturer’s protocol. Briefly, fibroblasts were cultured to 80–90% confluence, trypsinized, and resuspended in nucleofection solution with the siRNA [either negative controls (scrambled) or IL-1α or TGF-β1 siRNA (Santa Cruz Biotechnology, Inc.)]. For the validation of the assay, the test values from all 4 negative siRNA (Santa Cruz Biotechnology, Inc.) controls must be in the range of 1× standard deviation (±SD) of the means from 4 untreated cell controls. The cells were electroporated using the Amaxa nucleofection device. For each transfection, 1.5×10^6^ cells were electroporated with either 2 µg of the negative control, IL-1α or TGF-β1 siRNA. After the transfection, the fibroblasts were resuspended in RPMI media (GIBCO, Carlsbad, CA), counted with Trypan blue exclusion, and incubated at 37°C for 10 min. An appropriate number of cells were plated in wells of a 6-well plate and then incubated for an additional 48 h in normal culture medium (described above) prior to use.

### Statistical Analysis

All results are presented as means with standard errors unless otherwise stated. ANOVA and Student’s t tests were used for the comparison of means, and the z-test was used to assess the correlation analysis. P values less than 0.05 were considered statistically significant. SAS Statview software (SAS, USA) was used for data analysis.

## Results

### The Growth of Keratinocytes and Fibroblasts in Co-culture

**Figure 1:**
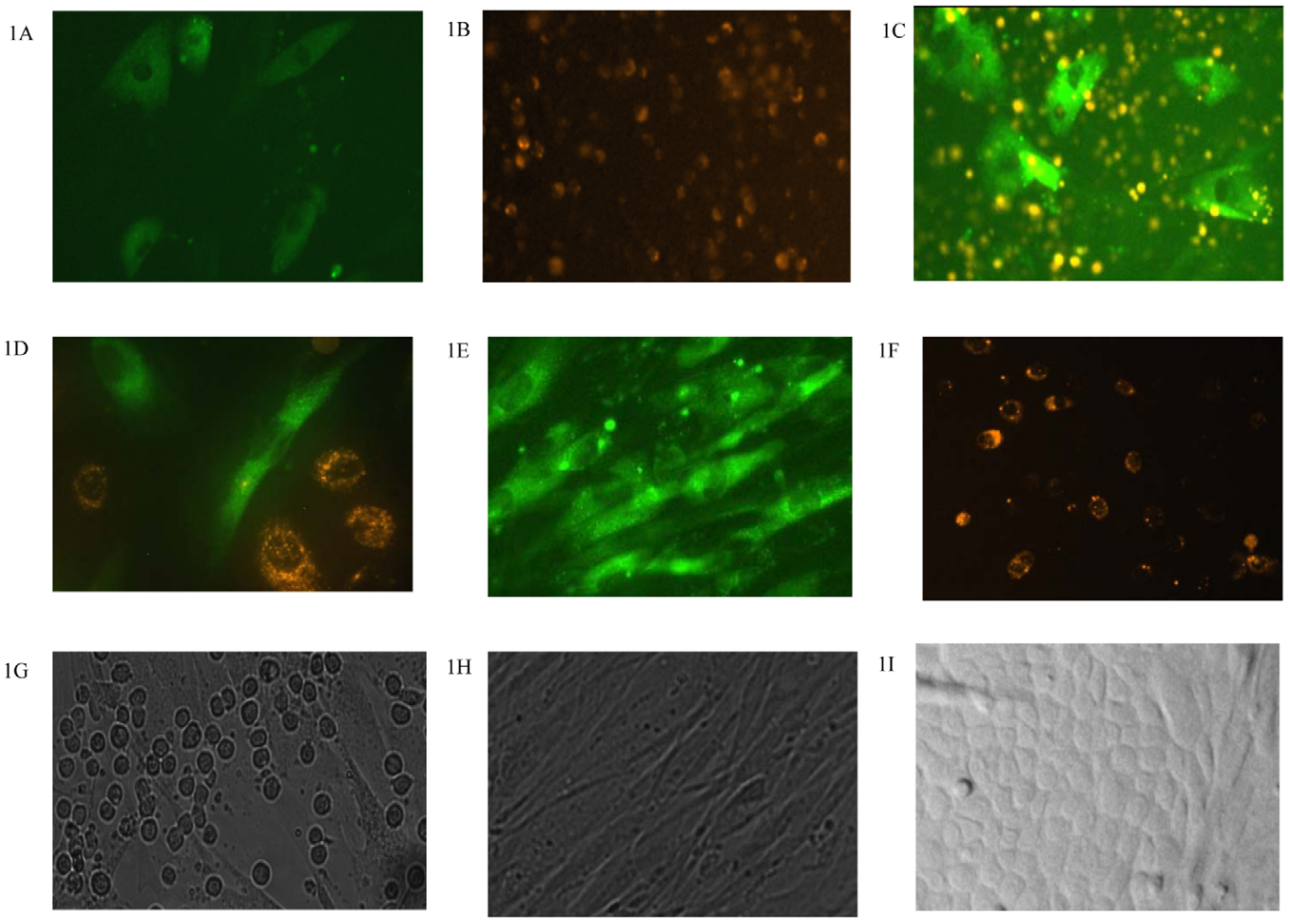
Keratinocytes and fibroblasts in cultures. 3×10^5^ fibroblasts, stained with PHK2 (green), were cultured in 6-well pre-coated plate for 48 hours (1A and 1C), and then 1×10^6^ keratinocytes, stained with PHK26 (orange), were seeded into the wells with (1C) or without (1B) fibroblasts. After 2 days, keratinocytes were also attached on the bottom of the well (1D). On day 10, fibroblasts tended to cover the entire bottom of the well (1E) but keratinocytes did not proliferate very much (1F). In the co-culture, significantly more keratinocytes (round cells) were growing on the fibroblast layer (1G). On day 15, the overgrowing fibroblasts resulted in some cells grew on others (1H), while keratinocytes could still form the skin-like layer on the top of fibroblasts (1I). 1A–1F were under fluorescence microscope (x200 or x400), 1G, 1H and 1I were under regular light microscope (x200), and natural light were used for 1I(x200).

To distinguish keratinocytes from fibroblasts in the co-culture, keratinocytes were stained with PHK26 and fibroblasts were stained with PHK2. The fibroblasts (3.0×10^5^/well), stained with PHK2 (green) were cultured in pre-coated (Collagen I/fibronectin/BSA), 6-well plates for 48 hours (Fig. 1A, C), and then keratinocytes (1×10^6^/well), stained with PHK26 (orange) were seeded into wells with (Fig. 1C) or without (Fig. 1B) fibroblasts. Keratinocytes attached to the bottom of the well by 2 days post-seeding (Fig. 1D). After 10 days in culture, the fibroblasts were completely confluent (Fig. 1E and G), and the keratinocytes tended to cover the fibroblasts to form skin-like structures (Fig. 1G). In the first 10 days of the culture, fibroblasts (Fig. 1A, C–E) proliferated faster than the kerotinocytes (Fig. 1B–D, F), although keratinocytes were seeded at a higher density. We also observed that the keratinocytes cultured with the fibroblasts (Fig. 1G) had a higher density than those cultured alone (Fig. 1F). On day 15 after the cultures were started, the fibroblasts overgrew and formed several layers of cells when grown alone (Fig. 1H); however, in the wells where the cells were co-cultured together, keratinocytes still covered most of the fibroblasts (Fig. 1I).

### Interactions between Keratinocytes and Fibroblasts in the Co-culture

**Figure 2:**
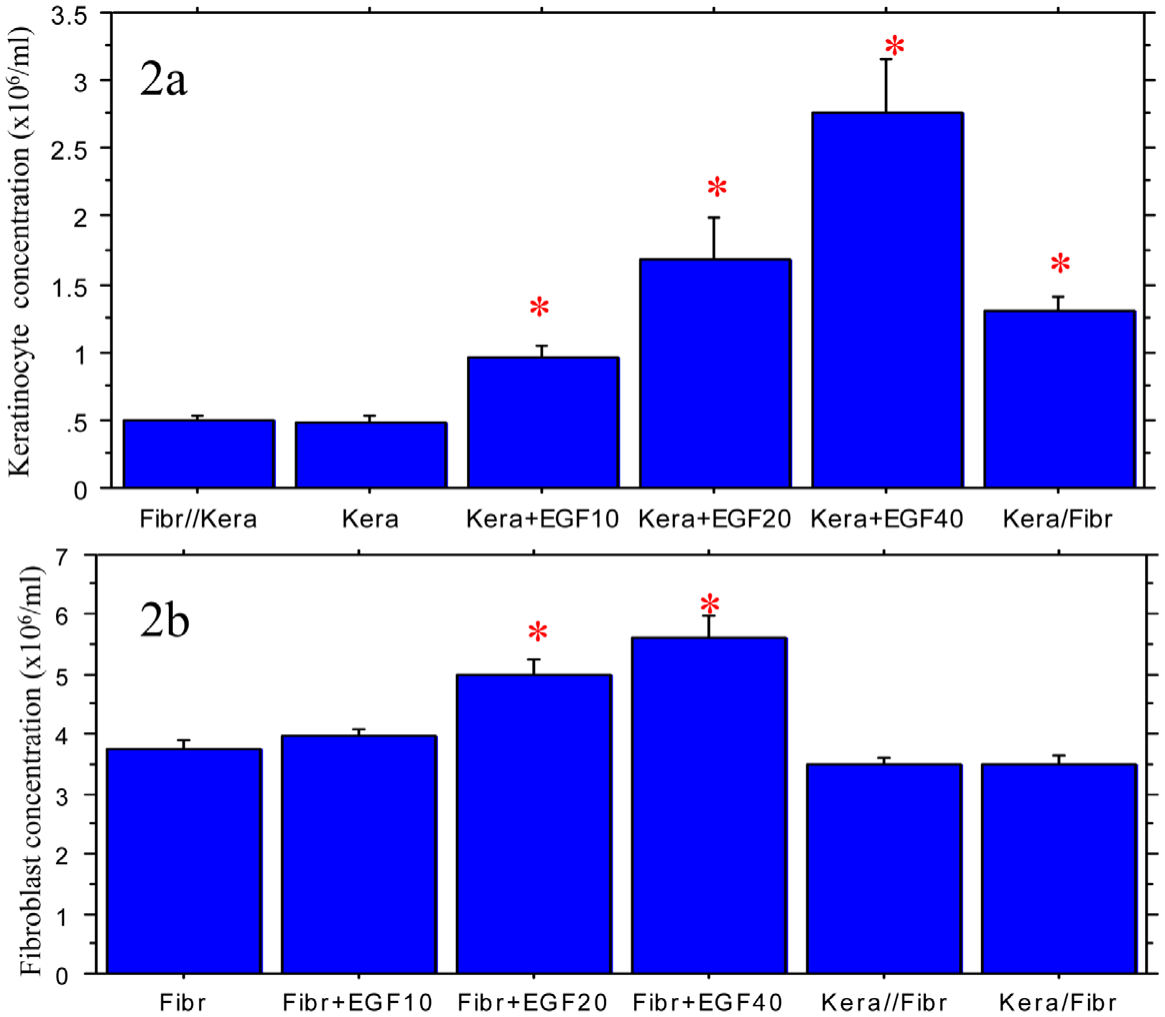
Effects of different cultures on the growth of keratinocytes (2A) and fibroblasts (**2B**)**.** 2A: Keratinocytes were cultured with fibroblasts in common 6-well plates (Kera/Fibr), with Fibr in transwell toseparate keratinocytes and fibroblasts (Kera//Fibr), with different concentrations of EGF (EGF10: EGF 10 ng/ml, EGF20: EGF 20 ng/ml and EGF40: EGF 40 ng/ml) or keratinocytes alone (Kera). Compared to Kera or Fibr//Kera, *P<0.01. Four cultures in each condition. 2B: Fibroblasts were cultured with keratinocytes in common 6-well plates (Kera/Fibr), with keratinocytes in transwell to separate keratinocytes and fibroblasts (Kera//Fibr), with different concentrations of EGF (EGF10: EGF 10 ng/ml, EGF20: EGF 20 ng/ml and EGF40: EGF 40 ng/ml) or fibroblasts alone (Fibr). Compared to the groups without star, *P<0.01. Four cultures in each condition.

Keratinocytes were cultured in the following conditions: with fibroblasts in common 6-well plates (Kera/Fibr), with fibroblasts in transwell plates (Kera//Fibr), with varying concentrations of EGF (Ker + EGF), or cultured alone (Kera). On day 5 after the experiment was initiated, the cells were trypsinized and counted by Trypan blue exclusion and flow cytometry. The keratinocyte cell count in the Kera/Fibr was significantly higher (P<0.05) than Kera//Fibr and Kera, suggesting that the increase in the number of keratinocytes in the early stage of the culture is secondary to the contact between keratinocytes and fibroblasts, although such diverges cannot be seen at later time points. To mimic the effects of fibroblasts on the keratinocytes, different concentrations of EGF were added to the keratinocyte cultures. Similar to the fibroblasts, EGF promoted keratinocyte growth and exhibited a dose-dependent effect (fig. 2a).

The number of fibroblasts also increased when EGF was applied to the cultures, but they were much less sensitive than keratinocytes to the effects of EGF. However, compared to the culture of fibroblasts alone, the fibroblasts did not exhibit an increase in growth when grown with keratinocytes, suggesting the fast growing fibroblasts cannot benefit from the co-culture with keratinocytes. In fact, the fibroblasts cultured with keratinocytes in both the 6-well and transwell plates had a slightly lower cell number compared to the group without keratinocytes, though the differences were not statistically significant (P>0.05). The number of fibroblasts also increased when EGF was applied to the cultures, but they were much less sensitive than keratinocytes to the effects of EGF. These results suggested that keratinocytes have a limited effect on the growth of fibroblasts, and that EGF promotes fibroblast proliferation but is not required for fibroblast proliferation (fig. 2b).

**Figure 3:**
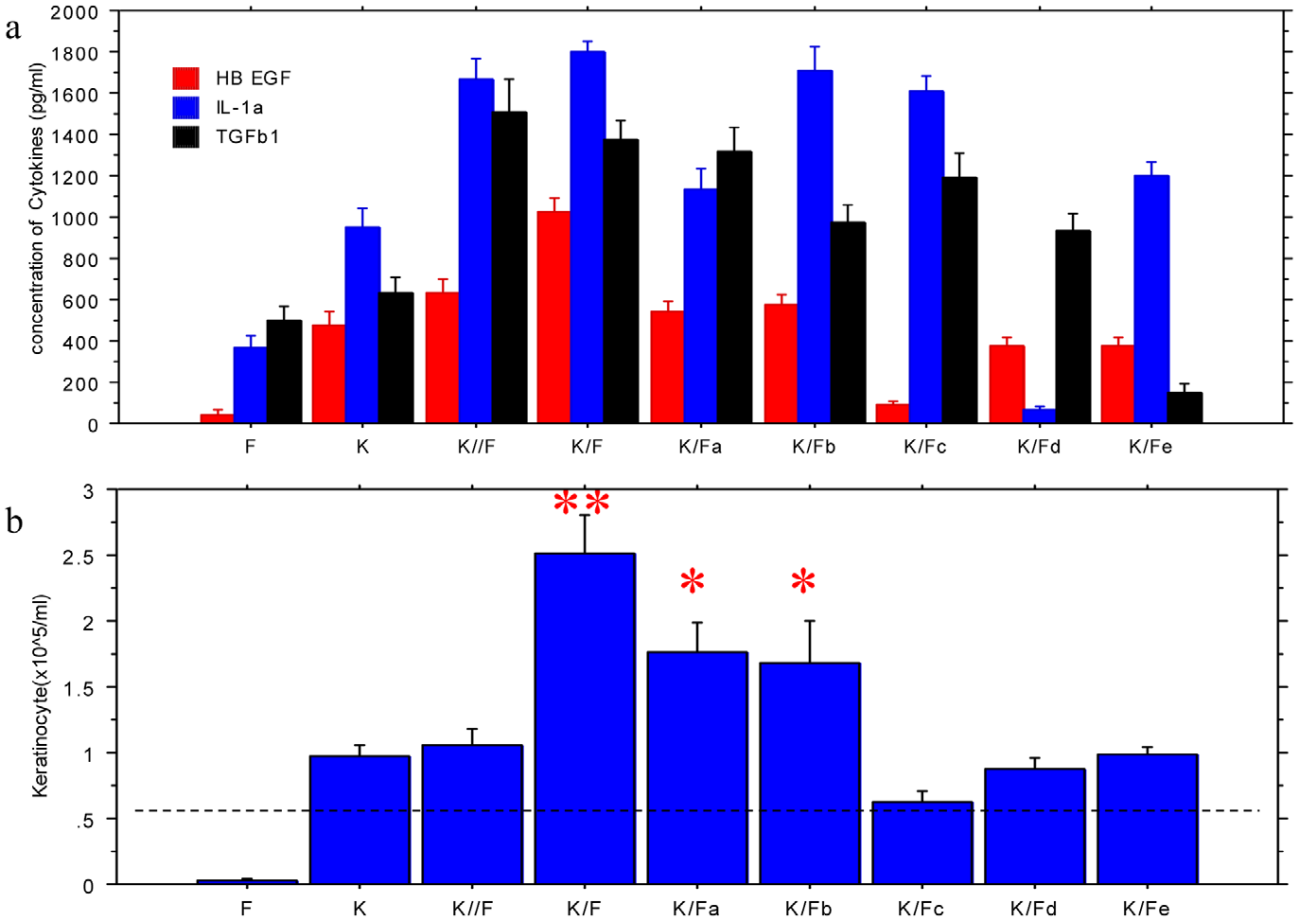
HB EGF, IL-1α and TGFβ1 levels (3a) as well as keratinocyte concentrations (3b) in different cell cultures. 3×10^5^ fibroblasts and 106 keratinocytes, respectively stained with PHK2 and PHK26, were cultured or co-cultured for 5 days. The supernatant was collected (Fig. 3a) and HB EGF, IL-1α and TGFβ1 levels were measured with ELISA. Keratinocytes (K) were harvested and resuspended in 2 ml media, and then were counted by Trypan blue exclusion and Flow Cytometry (Fig. 3b). K/F: keratinocytes were cultured with fibroblasts in common 6-well plates; K: keratinocytes alone; F: fibroblasts alone; In the co-cultures, keratinocytes were cultured with fibroblasts in transwells to separate keratinocytes and fibroblasts (K//F), with fibroblasts transfected with IL-1α siRNA (K/Fa) or TGFβ1 siRNA (K/Fb), or with fibroblasts and anti-HB EGF (K/Fc), anti-IL-1α (K/Fd) or anti-TGFβ1 (K/Fe). *P<0.05 and **P<0.01 compared to the groups without star. Four cultures in each condition. Dotted line shows the keratinocyte seeding level.

Since the fibroblast-induced increase in keratinocyte proliferation was reminiscent of the observed effects of EGF, we initially hypothesized that the EGF secreted by fibroblasts was perhaps the driving force behind the increased proliferation. However, ELISA assays with pg level sensitivity failed to detect EGF in any of the cell cultures. Instead, HB-EGF, a member of EGF family, as well as IL-1α and TGFβ1 were found in all cell cultures with keratinocytes (Fig. 3). Moreover, in cultures seeded with the same number of fibroblasts, those transfected with IL-1α and TGFβ1 siRNA produced lower levels of IL-1α and TGFβ1, respectively. Interestingly, the expression of HB-EGF in these co-cultures was also significantly reduced (both P<0.05). In contrast, TGFβ1 expression in the culture with IL-1α siRNA-transfected cells and the IL-1α expression in the culture with TGFβ1 siRNA-transfected cells did not change compared to the corresponding co-cultures without siRNA-transfected cells. Since the fibroblasts did not produce a significant level of HB-EGF in our current studies, and we did not find any report showing that fibroblasts could secrete HB-EGF, the above results suggest that the siRNA-mediated reduction in the production of IL-1 and TGF resulted in a concomitant reduction of HB-EGF in the keratinocytes co-cultured with those fibroblasts. The antibody neutralization tests for these cytokines further demonstrated the effects of IL-1α and TGFβ1 on HB-EGF levels. Moreover, the neutralization of any one of these cytokines with the respective antibody led to a decrease in the levels of the other two cytokines, with the effects of IL-1α and TGFβ1 neutralization on HB-EGF level being much greater than the effects of HB-EGF neutralization on IL-1α and TGFβ1 levels. These findings suggest that HB-EGF functions downstream of IL-1α and TGFβ1 in this system. As shown in Figure 3A and B, we found that (1) in all keratinocyte/fibroblast co-cultures without transwells (K/F), the keratinocyte concentrations correlated with HB-EGF levels (r = 0.815; P<0.01) better than IL-1α and TGFβ1 levels (P>0.05), (2) the keratinocytes in the co-culture without transwells proliferated significantly faster (P<0.01) than those in transwells (K//F), and (3) the inhibitory effects of siRNA transfection on IL-1α and TGFβ1 productions in fibroblasts led to a reduction (P<0.05)in HB-EGF production and keratinocyte proliferation, but more significant reductions were observed in the antibody neutralization tests (P<0.01), especially with the anti-HB-EGF antibody. These results suggested that the keratinocyte proliferation that was enhanced by co-culturing the cells with fibroblasts was mainly mediated by the cytokines produced by both cells, in which HB-EGF might play a central role in stimulating keratinocyte growth and could be up-regulated by IL-1α and TGFβ1 produced by fibroblasts.

In addition, we also found that the IL-1α expression level in the culture with TGFβ1 siRNA- transfected fibroblasts and the TGFβ1 expression level in the culture with IL-1α siRNA- transfected fibroblasts did not decrease, while the IL-1α expression level in the culture with the anti-TGFβ1 antibody and the TGFβ1 expression level in the culture with anti-IL-1α antibody were all significantly lower than those in the K/F group (Fig. 3A). These results suggested that a potential mutual regulation between IL-1α and TGFβ1 exists when fibroblasts and keratinocytes are cultured together.

**Figure 4:**
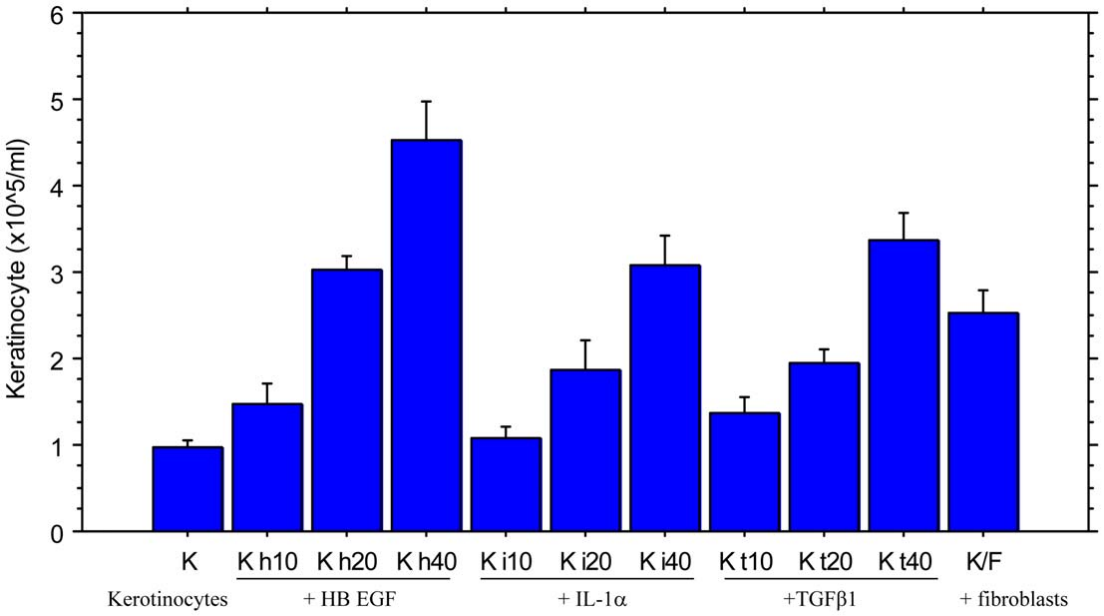
Effects of different cytokines on the growth of Keratinocytes. Keratinocytes and fibroblasts were respectively stained with PHK26 and PHK2 before co-culture. Keratinocytes were cultured with fibroblasts (K/F), or with 10 ng/ml, 20 ng/ml and 40 ng/ml of HB EGF (K h10, K h20 and Kh40), IL-1α (Ki10, K i20 and K i40) or TGFβ1 (K t10, K t20 and K t40), or keratinocytes alone (K). 6-well-plates were used in the culture and cells were resuspended in 10 ml after harvested on day 5 following the culture. Four cultures in each condition. Keratinocytes were counted by Trypan Blue exclusion and Flow Cytometry.

Since the fibroblasts did not produce significant levels of HB-EGF in the experiments described above (Fig. 3A), IL-1α and TGFβ1 or other cytokines most likely play a critical role in fibroblast-stimulated keratinocyte proliferation. However, the levels of IL-1α and TGFβ1 where the cells were co-cultured in the transwell were similar to the levels when the cells were cultured in direct contact with each other. Moreover, the stronger keratinocyte proliferation induced in co-cultures required direct contact between keratinocytes and fibroblasts. To further determine the roles of these two cytokines as well as the effect of cell-to-cell contact on keratinocyte proliferation, keratinocytes were cultured in the presence of different concentrations of HB-EGF, IL-1α and TGFβ1 and then compared to the keratinocytes/fibroblasts co-culture. Fig. 4 shows that all three cytokines stimulated kerotinocyte proliferation in a dose-dependent manner, but the level of cytokine required to reach the same level of growth stimulation found in the co-cultures was as much as 10 to 20-fold higher (15–30 ng/ml) than what was found in the co-culture media (1–2 ng/ml). Therefore, it is possible that the local concentrations of these cytokines in the cell-to-cell contact micro-environment are much higher than the overall levels in the media, which may be sufficient to affect proliferation.

**Figure 5:**
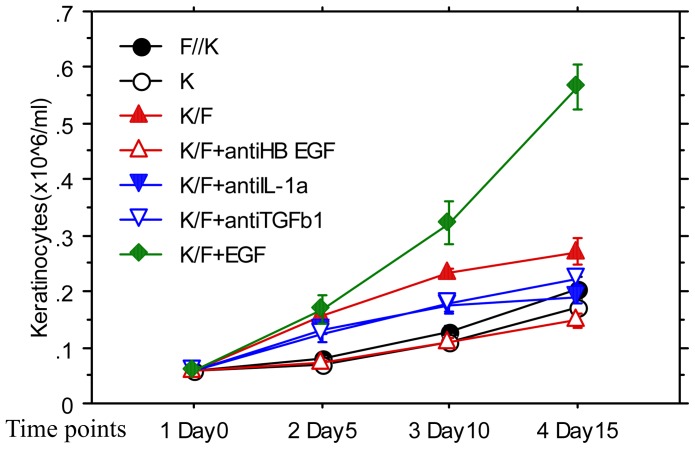
Effects of co-cultures on the growth of Keratinocytes in long term culture. Keratinocytes were cultured with fibroblasts in common 6-well plates (K/F), with Fibroblasts in transwell to separate keratinocytes and fibroblasts (K//F), with 20ng/ml EGF (K+ EGF), with fibroblasts and anti-HB EGF (K/F + anti-HB EGF), anti-IL-1α (K/F + antiIL-1a) or anti-TGFβ1 (K/F antiTGF β1) or keratinocytes alone (K). 6-well-plates were used in the culture and cells were resuspended in 12 ml after harvested. Four cultures in each condition. Keratinocytes and fibroblasts were respectively stained with PKH26 and PKH2 before culture and counted at the end of each culture (stage) by Trypan Blue exclusion and Flow Cytometry.

To understand the long-term effects of cytokines and co-culture on the proliferation of keratinocytes, some cultures were observed on the 5^th^, 10^th^, and 15^th^ day after the cultures were initiated. As shown in Fig. 5, the keratinocytes in the co-culture with additional EGF maintained fast proliferation after day 5, while those in other cultures grew much slower. The keratinocytes in the K/F group exhibited a marked reduction in the proliferation rate following day 10. Since the keratinocytes in the culture with EGF did not exhibit a reduction in the proliferation rate, the slower keratinocyte proliferation in the K/F group during the late stage of the co-culture could not be caused by an insufficient level of basic nutrition in media.

**Figure 6:**
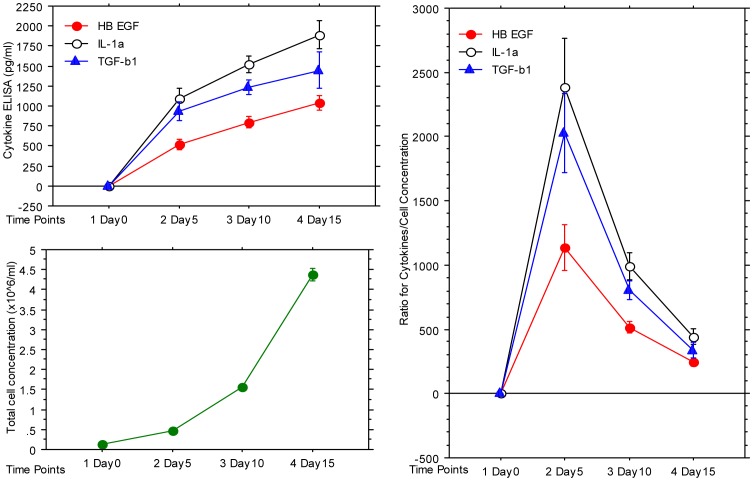
The relationships between cell concentration and HB-EGF, IL-1α, and TGF-β1 concentrations. Fibroblasts (3.0×10^5^ cells) and keratinocytes (1.0×10^6^ cells) were cultured in 6-well plates. The supernatant and cells that contained both keratinocytes and fibroblasts from 4 wells were harvested at each time point. The cells were then resuspended in 5 ml for counting. The cytokine concentrations were measured by ELISA. (6A) HB-EGF, IL-1α, and TGF-β1 concentrations at different time points. (6B) Total cell concentration (containing both keratinocytes and fibroblasts) at different time points. (6C) The ratio of HB-EGF, IL-1α, and TGF-β1 concentrations over the total cell concentration (×10^6^/ml) at different time points.

To further investigate if the reduction in the keratinocyte growth rate during the different stages of the culture was related to the levels of HB-EGF, IL-1α, or TGF-β1, the levels of these cytokines in the different cultures at different time points and the relationship between cell number and concentration of these cytokines were plotted (Fig. 6A–C). All cytokine levels (Fig. 6A) and total cell concentrations (keratinocytes + fibroblasts; Fig. 6B) increased steadily during the culture time period, but the cell concentration doubled faster than the concentration of all three cytokines after day 5, which resulted in a quick reduction in the cytokine/cell ratio during the late stage of the culture (Fig. 6C). The reduction in the concentration of cytokine per cell might explain the reduction in the keratinocyte growth rate during the late stage of the co-culture. When the data were analyzed together, we found that the fibroblasts grew faster than the keratinocytes, although the latter had a much higher cell seeding density. These data suggested that the reduction in keratinocyte proliferation in the co-cultures was at least partially due to the decreasing cytokine concentrations per cell, since the production of cytokines in the culture was slower than the proliferation of cells.

To understand how keratinocytes and fibroblasts interact during migration, several models have been developed [26], [27]. These models have employed similar methods of measuring the distance [26] of migration or migrating cell numbers [27], [28]. In our present model, coverslips with pre-seeded cells were placed in cell culture dishes or 6-well plates and then removed at the end of the culture. The cells that migrated off of the coverslips were removed by trypsinization and counted by Trypan blue exclusion and flow cytometry. This model allowed us to accurately determine the number of the cells affected by experimental factors at each time point.

**Figure 7:**
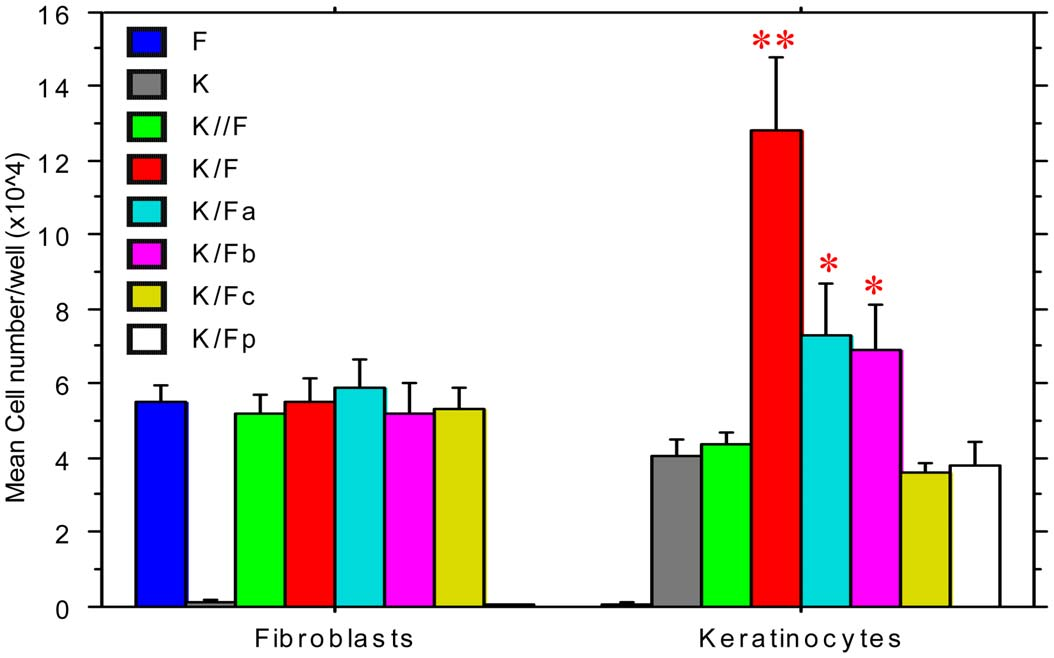
The effects of co-culture on the migration of keratinocytes and fibroblasts. Keratinocytes (1.0×10^6^ cells) were cultured with pre-seeded 3×10^5^ fibroblasts in common 6-well plates (K/F), with fibroblasts in a transwell to separate keratinocytes and fibroblasts (K//F), or with fibroblasts and anti-IL-1α (K/Fa), anti-TGFβ1 (K/Fb), or anti-HB EGF (K/Fc). Keratinocytes alone (K) and fibroblasts alone (F) were cultured as controls. All cells were treated with Mitomycin C and the fibroblasts in K/Fp were also treated with 1% paraformaldehyde PBS. 2 days after keratinocytes were seeded, the coverslips were transferred to new wells for additional analysis. The 6-well-plates containing cells on coverslips were used for additional analysis. After culturing the cells for 5 days, the coverslips were removed and the cells in the wells were trypsinized and resuspended in 1 ml for counting. Six cultures were analyzed for each condition. Keratinocytes and fibroblasts were stained with PKH26 and PKH2, respectively, before culturing the cells, and then counted by Trypan Blue cell exclusion and flow cytometry after culture. *P<0.05 and **P<0.01 compared to the group without the star. Between *and **, P<0.05.

As shown in Fig. 7, significantly more keratinocytes co-cultured with fibroblasts migrated off of the coverslips than the keratinocytes in the transwell controls (P<0.01), kerotinocytes alone control (P<0.01), and keratinocytes co-cultured in the presence of anti-IL-1α (P<0.05), anti-TGF-β1 (P<0.05), or anti-HB-EGF (P<0.01) antibodies. In contrast, the migration of fibroblasts was unaffected by any experimental factor. These results suggest that fibroblast-produced cytokines can enhance keratinocyte migration when the two cell types are in contact, possibly due to the effects of IL-1α and TGF-β1 produced by fibroblasts at cell contact points [29], where the fibroblasts may have accessibility for the delivery of cytokines to keratinocytes. The data also suggested that these two cytokines produced by fibroblasts may increase the migration activity of keratinocyes directly or/and indirectly by up-regulating HB-EGF.

## Discussion

The dynamics of fibroblasts and keratinocytes in co-culture have been studied by several investigators to illustrate the interaction between these two cell types [30]–[36]. When keratinocytes were overlaid on fibroblasts, a skin-like structure formed in some studies [37]–[39]. A similar result was obtained in our current study. Microscopic observation of keratinocytes and fibroblasts in co-culture did not show any obvious alterations in morphology, which indicated that the co-culture conditions in our study were suitable for both cell types.

In previous studies [2], [5], [40], it has been shown that the activities of both fibroblasts and keratinocytes are down-regulated at the end of wound healing, and that fibroblast activity can be further suppressed artificially. However, such down-regulation was not evident in our study. In contrast, only keratinocytes exhibited a reduction in the growth rate during the late stage of the co-culture. In the early stage of co-culture, fibroblasts significantly enhanced the proliferation of keratinocytes. Furthermore, this enhancement was related to the contact between these two cell types, and was abolished when these two cell types were separated by transwell inserts. Since EGF has been widely studied in the clinical setting as an enhancer of wound healing and has been found to inhibit scar formation [41]–[45], we hypothesized that the up-regulatory effects of fibroblasts on keratinocyte proliferation might be mediated by EGF produced by fibroblasts. Although the effects of the fibroblasts on kerotinocytes were reproduced by adding EGF to the culture media, examination of co-culture samples showed no detectable levels of EGF by ELISA. These results led us to examine other members of the EGF family.

Many cytokines are involved in wound healing [2], [46]. Heparin-binding EGF-like growth factor (HB-EGF) is a member of the EGF family of proteins [47]. It has been shown to play an important role in wound healing, cardiac hypertrophy, heart development and heart function [48]–[50]. Shirakata et al. demonstrated that HB-EGF is the predominant growth factor involved in epithelialization in skin wound healing *in vivo*, and that it functions by accelerating keratinocyte migration rather than proliferation [49]. Moreover, the studies of Hashimoto et al. suggested that HB-EGF is an autocrine growth factor for human keratinocytes, and HB-EGF and TGF-alpha act not only through an auto-inductive mechanism, but also by mutual amplification [51], [52]. Marikovsky et al. found that wound fluid-derived HB-EGF is synergistic with insulin-like growth factor-I for Balb/MK keratinocyte proliferation [53], [54]. Consistent with this study, we also observed that the keratinocytes under a proper dosage of irradiation stopped proliferating but could still produce higher level of HB EGF with IL1α or/and TGF β 2 stimulations, in which HB EGF levels were higher than non-stimulation but lower than non-irradiated cells with stimulation.

IL-1α and TGFβ1 are cytokines that have been extensively studied for their roles in keratinocyte-fibroblast interactions [34], [36], [55]–[59]. However, it is not clear yet how IL-1α and TGFβ1 regulate HB-EGF in kerotinocyte proliferation and migration promoted by the co-culture with fibroblasts. In our current studies, the proliferation of keratinocytes that were in contact with fibroblasts in the early stage of the co-culture was significantly enhanced compared to cultures containing only keratinocytes. In addition, this enhancement was not seen in transwell cultures or in cultures containing an anti-HB-EGF antibody, and was remarkably reduced in the cultures with anti-IL-1α or anti-TGFβ1 antibodies. When IL-1α siRNA or TGFβ1 siRNA-transfected fibroblasts was used in the co-culture, the enhancement of keratinocyte proliferation was also significantly reduced, since the production of IL-1α and TGFβ1 by fibroblasts was inhibited. HB-EGF was also reduced in cultures with IL-1α or TGFβ1 siRNA-transfected fibroblasts and in co-cultures with anti-IL-1α or anti-TGFβ1 antibodies, indicating that the keratinocyte proliferation upregulated by IL-1α and TGFβ1 in the co-culture was partially mediated by HB-EGF. The effects of the anti-HB-EGF antibody on keratinocyte proliferation were significantly greater than those of the anti-IL-1α or anti-TGFβ1 antibodies, suggesting that HB-EGF plays a central role in the regulation of kerotinocyte growth in these co-cultures. Since siRNA can only partially or/and transiently block target cytokine production and the inhibition antibodies we used in our experiments were all over-dosage, the inhibitory effects of siRNA was always weaker than those of inhibition antibodies in our observations.

After the early stage of the co-culture, the keratinocytes in all of the culture conditions proliferated at similar rates, with the exception of the EGF control (20 µg/ml of HB EGF was also used as the positive control and it led 4-fold stronger cell proliferation). The EGF culture showed faster late stage growth than the other cultures, suggesting a reduction of cell growth factors in these cultures. However, when the concentrations of the HB-EGF, IL-1α, and TGFβ1 were examined, it was found that they continued to increase as the cells grew. Further analysis into the ratio of HB-EGF levels and cell count revealed that the increase in HB-EGF, IL-1α, and TGFβ1 levels failed to match the growth rate, resulting in lower levels of these cytokines per cell during the later stage of the culture.

Both keratinocytes and fibroblasts have very high motility in culture [60]–[69], which enables them to meet and form cell-to-cell contacts even when co-cultured at very low densities. To confirm the effects of cell contact on keratinocyte proliferation and migration that was promoted by fibroblasts and relevant cytokines, transwell plates were used to separate the two cell types in this study. The two cell types were separated into the upper and lower chambers of the transwell to eliminate direct contact with each other, and the cell type being observed was placed in the lower chamber. The data clearly showed that without direct contact, the fibroblasts and the IL-1α and TGFβ1 cytokines produced by the fibroblasts did not have a significant effect on the proliferation and migration of co-cultured keratinocytes. To further confirm the effects of IL-1α, TGFβ1 and HB-EGF on keratinocyte proliferation, these cytokines were added to keratinocyte cultures at different concentrations and compared to the kerotinocye/fibroblast co-culture. The results showed that all three cytokines were able to stimulate keratinocyte proliferation, but require a 10-fold higher concentration of the cytokines, which suggested that since the overall cytokine level of the culture was insufficient to account for the observed effects, direct contact may be required to provide a microenvironment with sufficient cytokine levels.

Cell death (necrosis and apoptosis) could be a factor that affects the evaluation of cell proliferation by cell counting. To eliminate the interference of cell death on our experimental results, we did Anexin V binding assay for some samples in addition to our routine Trypan Blue Exclusion test for all samples. Trypan Blue Exclusion was assessed on days 5, 10 and 15, when the cells were harvested. The percentages of Trypan Blue-stained cells were less than 2% on days 5 and 10, and less than 5% on day 15 in all experiments. Since those auto-detached cells might be the main factor affecting the evaluation of the cell proliferation, the numbers of floating cells at different time points were examined and correlated to those of the total cells and the live cells very well (R^2^ = 0.922−0.978, p<0.001−0.0005), and the percentages of floating cells in total cells were at a very narrow range (3.19–4.86%), demonstrating that the floating cells did not significantly affect the evaluation of cell proliferation. Annexin V binding assay is a very sensitive method for evaluating the very early stages of Apoptosis. Using the method described in our previous study [21], we examined the Annexin V- positive cells. Unlike the dead cell counting and floating cell counting, the kerotinocytes (22.4±3.7%, n = 6) had significantly higher Annexin V positive percentages compared to fibroblasts (17.3±2.9%, n = 6, P<0.05) on day 15 following the culture. In the co-culture, the Annexin V positive percentage (19.7±4.3%, n = 6) was slightly higher than the mean Anexin V- positive percentage (19.2±4.9%, n = 6) of fibroblasts and keratinocytes sole culture, but the difference is not significant (P>0.1). In contrast, Annexin V positive percentages in all cultures are quite similar and in a very narrow range (3.2–4.7% on day 5 and 5.1–6.9% on day 10) in established cultures. These data suggested that the increased kerotinocyte proliferation in our co-culture was not caused by the reduced cell death or/and apoptosis. Since the Annexin V-positive cells were increased quickly on day 15, it is possible that the more dead cells would be observed if the cells were cultured more than 15 days.

Although it has been well-demonstrated that both IL-1α, and TGFβ1 are HB EGF inducers, the increased HB EGF in the co-culture of this study could be also caused by the higher cell number as a result of the increased cell proliferation. Examining HB EGF production in the media from keratinocyte cultures and keratinocyte-fibroblast co-cultures revealed that the keratinocyte cell number in the single culture took 15 days to reach the level found in co-cultures after only 5 days. After the media was changed and the cells were further cultured for 24 hours, the HB EGF concentration (292±48 pg/ml, n = 4) in the single culture was only less than 60% of that (541±48 pg/ml, n = 4) in the co-culture. When IL-1α (10 µg/ml) or TGFβ1(10 µg/ml) was added into these two cultures, the 24-hour HB EGF production jumped to 416±51 pg/ml (n = 4) and 475±39 pg/ml (n = 4) in the single cultures, and 647±72 pg/ml (n = 4) and 696±39 pg/ml (n = 4) in co-cultures. These data further support that co-culture, IL-1α and TGFβ1 can affect HB EGF and the higher cell number is not required for the enhanced HB EGF production in our culture system. Even so, the effects of the stronger cell proliferation in co-culture on the enhanced HB EGF production are still not negligible.

Keratinocyte proliferation and migration are fundamentally important steps in the reepithelialization of skin wounds [70]. There are several existing models for the study of keratinocyte migration. These studies have focused on either the distance of migration or the number of cells mobilized during the migration [26], [27]. In the model we used in this experiment, the number of cells that migrated off of the coverslip was counted to provide an assessment of the overall magnitude of keratinocyte migration. This coverslip-based cell culture system is very easy to use and does not require any additional equipment. We compared this assay and another classic migration assay, scratch assay [16]–[20], and found that the results of these two methods correlated very well (R^2^>0.90, P<0.01). Using the coverslip assay, we demonstrated that the migration of kerotinocytes was enhanced by direct contact with fibroblasts, which correlated with the levels of HB-EGF, IL-1α, and TGFβ1 in the culture media. Moreover, this enhancement was inhibited by an anti-HB-EGF antibody and reduced by anti-IL-1α and anti-TGFβ1 antibodies when they were added to the cultures. These findings suggest that direct contact with fibroblasts is required to promote the migration of keratinocytes, and that HB-EGF, IL-1α and TGFβ1 play important roles in the interaction between keratinocytes and fibroblasts. Since mytomycin C treatment was used in the migration assay, one concern is if the mytomycin C- treated keratinocytes can still produce more HB-EGF in their responses to IL-1α and TGFβ1 stimulations. We examined the HB-EGF expression at both protein level (culture supernatant) by ELISA and mRNA level (cells) by real time reverse transcription PCR which was normalized by the housekeeping gene 36B4. On day 5 following IL-1α and TGFβ1 stimulations, mytomycin C- treated keratinocytes, similar (P>0.1) to untreated ones, produced a 3–4 folds more HB-EGF protein (p<0.05−0.01) and 5–8 folds more HB-EGF mRNA (P<0.01). This further supports that HB-EGF also plays an important role in increasing the keratinocyte migration in our co-culture system.

The literature is divided on the effects of TGFβ on the migration of kerotinocytes. One study showed TGFβ suppressed migration of keratinocytes [28], but consistent with most other studies [71]–[74], our data clearly demonstrated that both TGFβ1 and IL-1α produced by fibroblasts promoted the migration of keratinocytes. Since TGFβ has at least three subtypes (TGFβ1, TGFβ2, and TGFβ3), it is possible that the different subtypes of TGFβ have different functions. Moreover, a specific subtype of TGFβ may have been dominant in the mixed subtypes of TGFβ in the Tsuboi’s study. In addition, our data are also consistent with those from the studies by Koivisto et al., in which keratinocyte migration was shown to be induced by TGFβ1, EGF and other cytokines, and these inductions were promoted by autocrine HB-EGF [75]. In our study, the up-regulation of HB-EGF by IL-1α and TGFβ1 was further highlighted.

In summary, the up-regulation of HB-EGF by IL-1α and TGFβ1 appears to be a major driving force behind the effects of co-cultured fibroblasts on the proliferation and migration of keratinocytes. In addition, our data also support the therapeutic benefits of EGF on wound healing and scar formation.
